# Eye, Ear, Nose, and Throat

**Published:** 1896-02

**Authors:** 


### *SELECTIONS AND ABSTRACTS.*

---

### EYE, EAR, NOSE, AND THROAT.

EDITED BY DUNBAR ROY, A.B., M.D.

### WHAT SHALL A GENERAL PRACTITIONER DO FOR AN ACUTE OTITIS?

When the pain is due to inflammation either of the external auditory meatus or of the middle ear, and the patient is seen in the early stages of the attack, the physician should attempt to abort the inflammation as well as to relieve the symptoms. The most efficient measure for aborting the attack is undoubtedly the local abstraction of blood. In very young children either wet cupping or the use of natural leeches is difficult, although the cup can be applied if the child is firmly held; and if the physician does not possess enough confidence in himself to manipulate instruments in the external auditory canal, it is certainly wise to employ local depletion. The region selected for the application either of the wet cup or of leeches is that lying close to and immediately in front of the tragus. From half an ounce to one and a half ounces of blood may be withdrawn in this region, and in many cases the measure is beneficial. The patient should be kept in bed and a free catharsis instituted. If the case is seen very early, it is frequently wise to follow the abstraction of blood by the administration of an opiate sufficiently powerful to quiet the patient for five or six hours, in the hope that depletion and absolute rest for several hours may abort the attack. For the further relief of pain the use of heat about the ear is a means at the disposal of all, and is certainly valuable, either after local depletion or where this measure is not deemed advisable. The most convenient method of employing dry heat is by means of the Japanese pocket-stove; the hot-water bag, although less convenient, is equally efficacious. Where these are not available, a stove-lid, a flat-iron, or a brick previously heated, wrapped in a piece of flannel and placed under the pillow, will supply the place of the more convenient apparatus above described. In order that the heat may be applied more directly, the author occasionally recommends that a small hot salt-bag be introduced into the meatus, heat being applied externally by means of the hot-water bag or other device, as stated before. These salt-bags are conveniently made by cutting off the finger tips of a kid glove, filling the tips with salt, and placing them upon a hot plate until they are thoroughly heated, after which they are placed just within the meatus and heat is applied externally, as already described.

If there is one point we should emphasize more than another, it is that under no circumstances is any oily substance to be introduced into the meatus. The old practice of dropping warm sweet oil or a mixture of sweet oil and laudanum into the external auditory canal for the relief of otalgia is a relic of barbarism that deserves no place in modern medicine.

The writer does not favor the use of moist heat in the early stages of an acute otitis, consequently the irrigation of the canal with warm water, or the application of poultices to the ear, the canal being previously filled with water, are not measures to be advised. Moist heat softens the tissues and encourages their disintegration, while our efforts in the early stages should be directed towards aborting the inflammation. Heat is employed in the early stages simply for the relief of pain, and dry heat does not encourage suppuration, while moist heat certainly favors it.

If, after the effects of the opiate have disappeared, the pain reappears, it is unwise to administer a second dose, as it will only mask the symptoms. The local abstraction of blood and the application of dry heat are then the only resources at the command of the physician, previous to the appearance of discharge from the ear, unless he is able to use the head-mirror and inspect the parts affected.

After discharge has made its appearance, frequent irrigation of the external meatus by means of a weak antiseptic solution, such as a solution of the bichloride of mercury (1 to 5000) or of boracic acid, is the best measure for combating the inflammatory process and for preventing its extension to the neighboring bony parts. In many of these cases the discharge is serous in character, and will continue so unless it is infected, as it lies in the external auditory canal. All that is needed is to keep the meatus perfectly clean and prevent infection of the discharge. It is unwise to stop the meatus with cotton or to keep the ear covered, as in this way local infection of the canal is liable to occur, causing either circumscribed or diffuse inflammation. While the ear is discharging considerable relief may sometimes be obtained by filling the meatus with warm water and applying heat externally; a poultice covering the auricle, however, is not necessary, and causes so much maceration of the integument as to constitute a disadvantage; if the canal is filled with water and dry heat is applied externally, we have the beneficial action of the heat and moisture upon the parts affected without inflicting any injury upon the auricle.

After the acute symptoms have subsided, certain measures directed towards checking the discharge may seem necessary. It must be remembered that under no condition should any attempt be made to diminish the quantity of the discharge until the temperature becomes normal and all pain has disappeared. In the large proportion of cases where spontaneous rupture of the drum membrane has taken place, if the parts are kept carefully cleansed, in the manner previously described, the discharge will cease spontaneously; in a small proportion of cases it continues. The use of astringent instillations cannot be recommended. In cases where the discharge persists, it is perfectly safe to use a saturated solution of boric acid in alcohol as an instillation, after syringing. A few drops of this solution introduced into the meatus by means of the medicine-dropper, after careful syringing, may be employed with perfect safety, and is usually sufficient to stop the discharge. The objection to solutions of sulphate of zinc and other kindred instillations is that they form an excellent nidus for the development of vegetable parasites, and unless the physician can inspect intelligently, they possess no advantage over the alcoholic solution of boric acid.—DR. DENCH, *Archives of Pœdiatrics*, May, 1895.

